# Nutritional biomarkers regulating tumor immune microenvironment in osteosarcoma and as predictors of immune checkpoint inhibitor responses

**DOI:** 10.3389/fimmu.2026.1909913

**Published:** 2026-09-10

**Authors:** Haoyang Ren, Chengchun Shen

**Affiliations:** Department of Foot and Ankle Surgery, Ningbo Clinical Research Center for Orthopedics, Sports Medicine & Rehabilitation, Ningbo No.6 Hospital, Ningbo, China

**Keywords:** biomarker, ICIs - immune check point inhibitors, nutrition, osteosarcoma, TME

## Abstract

Osteosarcoma(OS) exhibits poor and heterogeneous responses to immune checkpoint inhibitors(ICIs), largely due to its highly tumor immunosuppressive microenvironment(TIME). This review explores how nutritional biomarkers regulate the OS tumor microenvironment(TME) and serve as predictors of ICIs efficacy. Key biomarkers, including prognostic nutritional index(PNI), vitamin D and IDO modulate tumor-infiltrating lymphocytes (TILs), CD8^+^ T-cell function, macrophage polarization, and cytokine–metabolite crosstalk through mechanisms such as nutrient depletion, lactate acidification, and inflammatory signaling. These biomarkers not only reflect host nutritional and inflammatory status but also actively shape an TIME that contributes to ICIs resistance. Targeted nutritional interventions and combinations with IDO show potential to reprogram the TME and enhance antitumor immunity. This review highlights the value of nutritional biomarkers for patient stratification, prognostic assessment, and the development of personalized nutrition–immunotherapy strategies, providing a foundation for overcoming treatment resistance in OS.

## 1 Introduction

Osteosarcoma (OS) is one of the most common primary malignant bone tumors. OS is largely refractory to conventional chemotherapy, and the emergence of immunotherapy has provided new therapeutic opportunities for OS patients. However, immunotherapy is not effective for all patients, and its therapeutic efficacy is closely associated with the infiltration and activation status of immune cells within the tumor microenvironment (TME) (1–3). OS is characterized by profoundly immunosuppressive tumor immune microenvironment, in which complex interactions among tumor cells, immune cells, and stromal cells promote immune evasion and limit the efficacy of immunotherapies targeting PD-1, CTLA-4, and other immune checkpoints (4, 5). Therefore, reversing immunosuppression and remodeling the tumor immune microenvironment are critical to improving the therapeutic efficacy of immunotherapy in OS. Among these immune components, tumor-infiltrating lymphocytes(TILs) are considered critical determinants of response to immunotherapy. Studies have shown that OS immunosuppressive microenvironment (TIME) is characterized by increased infiltration of immunosuppressive cells and functional exhaustion of effector T cells, resulting in an immunosuppressive phenotype that limits the efficacy of immunotherapy (6, 7). The formation of this immunosuppressive microenvironment is closely linked to nutritional and metabolic reprogramming. Through adaptive metabolic alterations, OS tumor cells not only meet their own energy and biosynthetic demands for rapid proliferation but also actively shape a nutritional imbalance, acidic, and highly TIME, thereby exacerbating the immunologically “cold” phenotype (8–10). This review focuses on the immunosuppressive features of the OS TME, the critical role of tumor cell nutritional and metabolic reprogramming in shaping the TIME. The aim is to provide theoretical support and potential intervention strategies for precision immunotherapy for OS.

In recent years, nutritional biomarkers have shown significant progress in predicting immune checkpoint inhibitors(ICIs) efficacy and modulating the TME. In OS, baseline PNI has demonstrated clear prognostic value (11, 12). Studies have shown that serum albumin levels and vitamin D status are key biomarkers for predicting responses to immunotherapy, and that exogenous vitamin D supplementation can reduce the incidence of immune-related adverse events (irAEs) (13–15). Furthermore, Wu et al. found that pathways involved in vitamin and cofactor metabolism were closely associated with the extent of immune cell infiltration in OS (16). By precisely regulating the TME, nutritional biomarkers are expected to be translated into personalized precision medicine interventions in the future, significantly improving ICIs efficacy and enhancing patient prognosis.

Previous reviews have predominantly focused on specific metabolic pathways, such as glucose metabolic reprogramming, or on individual nutritional factors, including dietary polyphenols and vitamin D, in the development and progression of OS (17–19). However, comprehensive syntheses of nutritional metabolic dysregulation and its interplay with the TME remain limited. Therefore, from the perspective of nutrition–metabolism–immune regulatory interactions, this review focuses on how nutritional and metabolic alterations in OS reshape the TME to exacerbate immune escape. It systematically explores how nutritional abnormalities promote immune escape by remodeling the TME and evaluates the potential value of related nutritional biomarkers in ICIs efficacy prediction. The goal is to provide a theoretical basis for developing individualized nutritional intervention and immunotherapy combination strategies, as well as theoretical and translational support for formulating personalized nutritional adjuvant treatment regimens to enhance long-term benefits from ICIs.

## 2 Mechanisms of nutritional biomarker regulation of the tumor immune microenvironment in osteosarcoma

**Figure 1:**
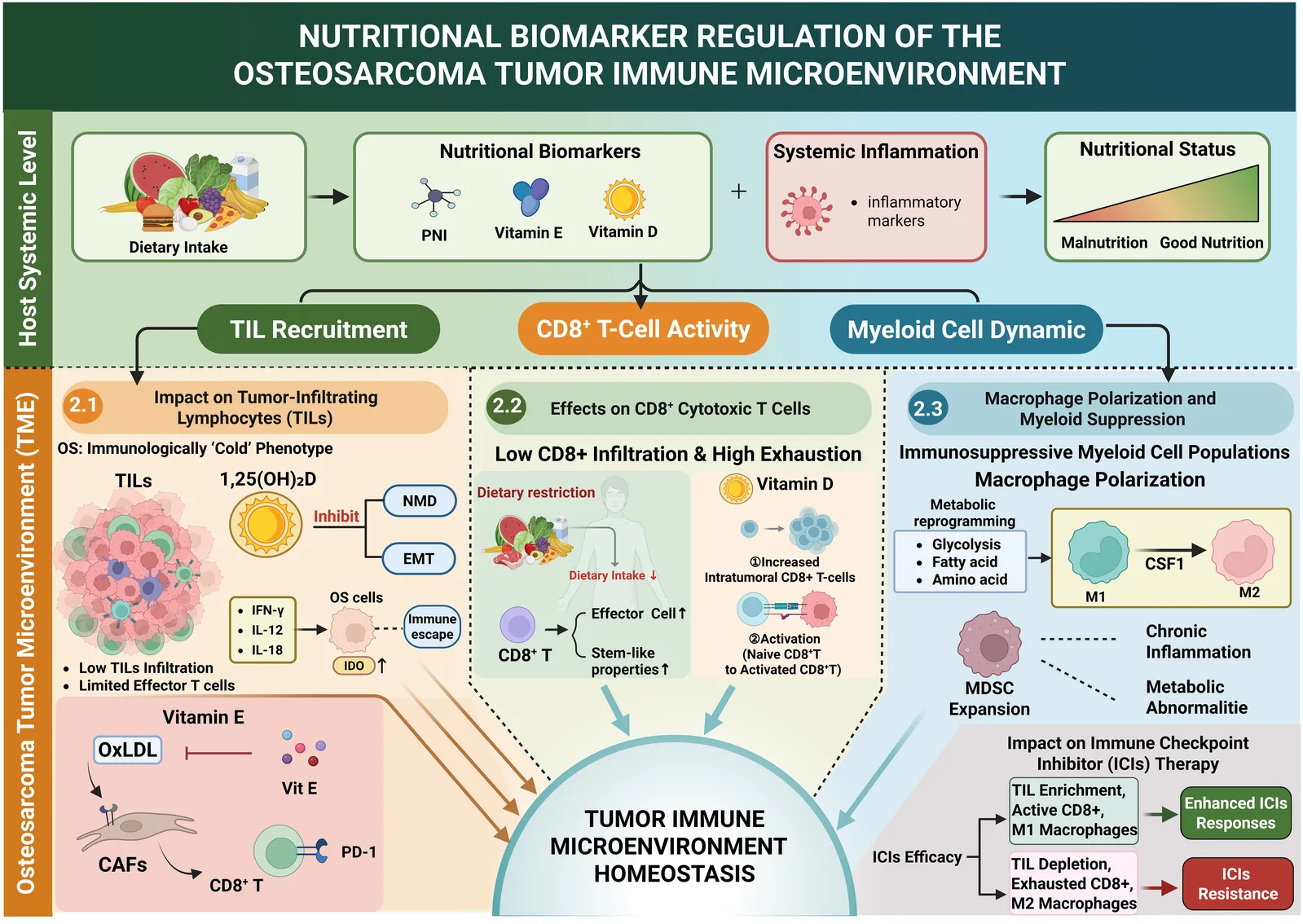
Nutritional regulation of the osteosarcoma tumor immune microenvironment. Nutritional status and related biomarkers, including PNI, vitamin D, and vitamin E, influence systemic inflammation and remodel the osteosarcoma TME by regulating TILs recruitment, CD8^+^ T-cell activity, and myeloid-cell polarization. (Created in BioRender).

Nutritional biomarkers not only reflect patients’ overall nutritional and inflammatory status but also directly or indirectly regulate TILs, CD8^+^ T cells, macrophage polarization. These regulatory mechanisms collectively influence the strength of ICIs-induced antitumor immune responses. This section analyzes the regulatory mechanisms of nutrition-related biomarkers on the homeostasis of the OS tumor microenvironment and explores their potential impact on ICIs therapy (**Figure 1**).

### 2.1 Impact on tumor-infiltrating lymphocytes

TILs represent a critical component of the TME and serve as a key indicator of host antitumor immune activity. The abundance, composition, and functional status of TILs reflect the immune landscape of tumors and are closely associated with the efficacy of immunotherapy. Emerging evidence suggests that tumor immunity is not solely determined by immune checkpoint signaling but is also profoundly influenced by systemic nutritional status and metabolic conditions. Tumor cell metabolic reprogramming can alter the metabolic pathways of key nutrients, such as glucose and glutamine, within the TME, thereby affecting the generation and consumption of related metabolites, ultimately reshaping the tumor microenvironment and regulating host immune responses (20–22). Metabolic competition between tumor cells and immune cells for limited nutrients within the TME can impair the survival and functional activity of immune effector cells, including TILs, leading to T-cell dysfunction, immune suppression, and reduced responsiveness to immunotherapy (23–25).

Vitamin D is an important regulator of T-cell function, and abnormalities in the vitamin D–vitamin D receptor (VDR) signaling pathway have been increasingly identified in various solid tumors. The dysregulation of vitamin D signaling in OS, characterized by altered VDR expression and serum vitamin D levels, has been implicated in tumor metastasis and aggressive biological behaviors, indicating that vitamin D status may represent a clinically relevant nutritional marker associated with immune modulation and disease progression (26–29). Karkeni et al. observed that vitamin D supplementation under standard diet conditions significantly increased intratumoral CD8^+^ T-cell infiltration in a breast cancer mouse model, enhanced T-cell activation, drove a shift from CD62L^+^ naïve to CD44^+^ activated/memory phenotypes, and elevated Granzyme B expression (27). Although direct data on TILs in OS are limited, Capobianco et al. demonstrated 1,25-dihydroxyvitamin D(1,25(OH)_2_D) suppresses OS progression by inhibiting nonsense-mediated mRNA decay(NMD) and epithelial–mesenchymal transition (EMT), thereby reducing tumor cell migration (30). Therefore, alterations in vitamin D status or VDR signaling may represent important nutritional determinants of antitumor immunity and could contribute to the heterogeneity of immunotherapeutic responses in OS.

As a nutrition-related metabolite, the tryptophan/IDO–kynurenine pathway significantly inhibits TILs infiltration and function by depleting dietary tryptophan and generating kynurenine. Liebau et al. found that IFN-γ, IL-12, and IL-18 induced IDO expression in OS cell lines, particularly in the presence of activated lymphocytes (31). This directly causes local tryptophan depletion and kynurenine accumulation, thereby suppressing T-cell proliferation and recruitment. This metabolic alteration may impair effector immune cell function, and contribute to immune escape. Hiroshi et al. reported that IDO positivity was frequently observed in OS and was associated with poorer progression-free survival(PFS) (32). Moreover, accumulating evidence suggests that combining IDO inhibitors with immune checkpoint inhibitors enhances antitumor immune responses across multiple tumor types (33). Farooq et al. further emphasized IDO as a novel immunotherapeutic target in OS, as its inhibition may suppress tumor metastasis and contribute to the restoration of TILs activity (34, 35).

Nutritional status influences lymphocyte generation and circulation. Clinical observations show that the PNI and systemic inflammatory markers correlate with immune status and prognosis in OS patients. Patients with low PNI are more likely to exhibit enhanced inflammatory responses and immunosuppressive microenvironments, suggesting an indirect association between nutritional status and TILs infiltration (36). The IGF-1/IGF-1R signaling axis is an important regulator of OS cell proliferation, survival, and metastasis, and its aberrant activation may also contribute to therapeutic resistance (37, 38). Recent evidence indicates that short-term fasting can enhance tumor sensitivity to PD-1 immune checkpoint blockade by reducing circulating IGF-1 levels (39). Caloric restriction has likewise been shown to suppress IGF-1-related signaling and exert antitumor effects (40). Increasing histone H3K27 acetylation in OS cells promotes the infiltration and activation of TILs and CD8^+^ T cells, providing a novel epigenetic strategy to enhance the efficacy of immunotherapy (41). This suggests that histone modifications may provide a novel therapeutic strategy for overcoming resistance to immunotherapy by remodeling the TME and promoting the infiltration and activation of TILs. Based on the available evidence, it may be speculated that dietary interventions aimed at reducing IGF-1 levels could, to some extent, modulate the OS TME and potentially enhance the antitumor efficacy of immunotherapy. However, this hypothesis is currently based primarily on findings from other tumor types and therefore requires further validation in OS-specific models and clinical studies.

### 2.2 Effects on CD8+ T cells

CD8^+^ T cells are the primary effector cells through which ICIs exert antitumor effects. In the OS tumor microenvironment, CD8^+^ T cell infiltration and activation are generally low, which is closely associated with limited immunotherapy efficacy (42–44). Sun et al. reported that macrophages and T cells constitute the predominant immune-cell populations within the OS microenvironment. Notably, CD4^+^ helper T cells were more abundant than effector CD8^+^ T cells, while the CD8^+^ T cell population exhibited an exhausted phenotype (45). Beyond these local immunosuppressive features, systemic nutritional and inflammatory status may further shape the functional fitness of CD8^+^ T cells. Dietary restriction (DR) enhances antitumor immune responses and delays tumor growth by increasing the abundance of CD8^+^ effector T cells and promoting their stem-like properties and metabolic adaptability (46).

Dysregulated lipid metabolism critically remodels the TME and drives immunosuppression and T cell dysfunction. A recent study reveals that in OS, oxidized low-density lipoprotein (OxLDL) reprograms fibroblasts to induce CD8^+^ T cell exhaustion via paracrine signaling. Scavenging microenvironmental OxLDL with vitamin E (VitE) disrupts this metabolic axis, remodels the TME, and significantly enhances intratumoral CD8^+^ T cell infiltration, offering a novel strategy for metabolic intervention to potentiate PD-1 immunotherapy (47). Amino acid metabolic reprogramming regulates tumor-cell proliferation and CD8^+^ T cell function by altering nutrient competition and metabolic states. Recent study has demonstrated that dietary methionine restriction or pharmacological targeting of methionine metabolism through MAT2A inhibition can reverse the TIME of MTAP-deficient OS by activating IKZF1-mediated PD-L1 expression, enhancing CD8^+^ T cell infiltration and antitumor immune responses, thereby improving sensitivity to immune checkpoint therapy (48). Similarly, tryptophan metabolism represents an important metabolic pathway regulating the tumor infiltration and effector functions of CD8^+^ T cells. Although direct evidence demonstrating that targeting tryptophan metabolism can effectively reshape the immune microenvironment of OS remains limited, previous studies have shown that modulation of tryptophan metabolism or inhibition of IDO activity may increase tumor-infiltrating CD8^+^ T cells and enhance their antitumor functions (49). Therefore, interventions targeting tryptophan metabolism and IDO inhibitors may represent potential strategies for sensitizing tumors to anti-PD-1 therapy. OS cells enhance aerobic glycolysis to provide the energy and metabolic intermediates required for rapid proliferation, invasion, and metastasis. Glycolysis-derived lactate and the resulting acidic microenvironment can further suppress CD8^+^ T cell infiltration and effector function through glucose competition, lactate accumulation, and immune checkpoint upregulation, leading to mitochondrial dysfunction, impaired cytotoxicity, and T-cell exhaustion and thereby promoting immune escape. Therefore, targeting glycolysis and its associated regulators, particularly P4HA1, may restore the antitumor activity of CD8^+^ T cells and enhance the sensitivity of OS to immunotherapy (50).

### 2.3 Macrophage polarization and myeloid suppression

Macrophages and myeloid-derived suppressor cells (MDSCs) constitute the major immunosuppressive cell populations in the OS microenvironment; their accumulation and functional states are closely associated with immune escape, disease progression, and treatment resistance (51–53). Single-cell RNA sequencing studies have revealed complex immunosuppressive myeloid cell populations in the OS microenvironment that suppress T-cell function and support tumor growth through multiple mechanisms (54, 55). Metabolic reprogramming of tumor-associated macrophages is a critical determinant of their polarization and function. Remodeling of multiple metabolic pathways, including glycolysis, fatty acid oxidation and the metabolism of amino acid, tryptophan, and glutamine, can regulate macrophage phenotype (56, 57). OS cells induce the polarization of bone marrow-derived macrophages toward an M2-like phenotype through the production of CSF1, whereas CSF1R inhibitors can reprogram macrophages and thereby suppress tumor progression and metastasis (58). Expansion of MDSCs is associated with chronic inflammation and metabolic abnormalities, both of which are typically exacerbated by declining nutritional status. Therefore, assessment of myeloid suppression in the OS immune microenvironment should incorporate host nutritional and inflammatory indicators to improve understanding of ICIs resistance mechanisms and enhance efficacy prediction (59).

In sum, nutritional biomarkers serve as important regulators linking systemic nutritional status, metabolic homeostasis, and local immune remodeling within the OS tumor microenvironment. Through modulation of nutrient availability, metabolic competition, and immune cell function, nutrition-related factors influence the infiltration, activation, and exhaustion of TILs and CD8^+^ T cells, while simultaneously shaping macrophage polarization and myeloid-mediated immunosuppression. Alterations in pathways involving vitamin D signaling, tryptophan metabolism, lipid metabolism, glycolysis, and amino acid metabolism may contribute to immune escape and reduced responsiveness to ICIs. Therefore, further studies integrating nutritional biomarkers with immune profiling and clinical immunotherapy outcomes are required to establish their predictive value and therapeutic potential in OS.

## 3 Nutritional biomarkers for personalizing ICIs treatment and optimizing prognosis in osteosarcoma

**Figure 2:**
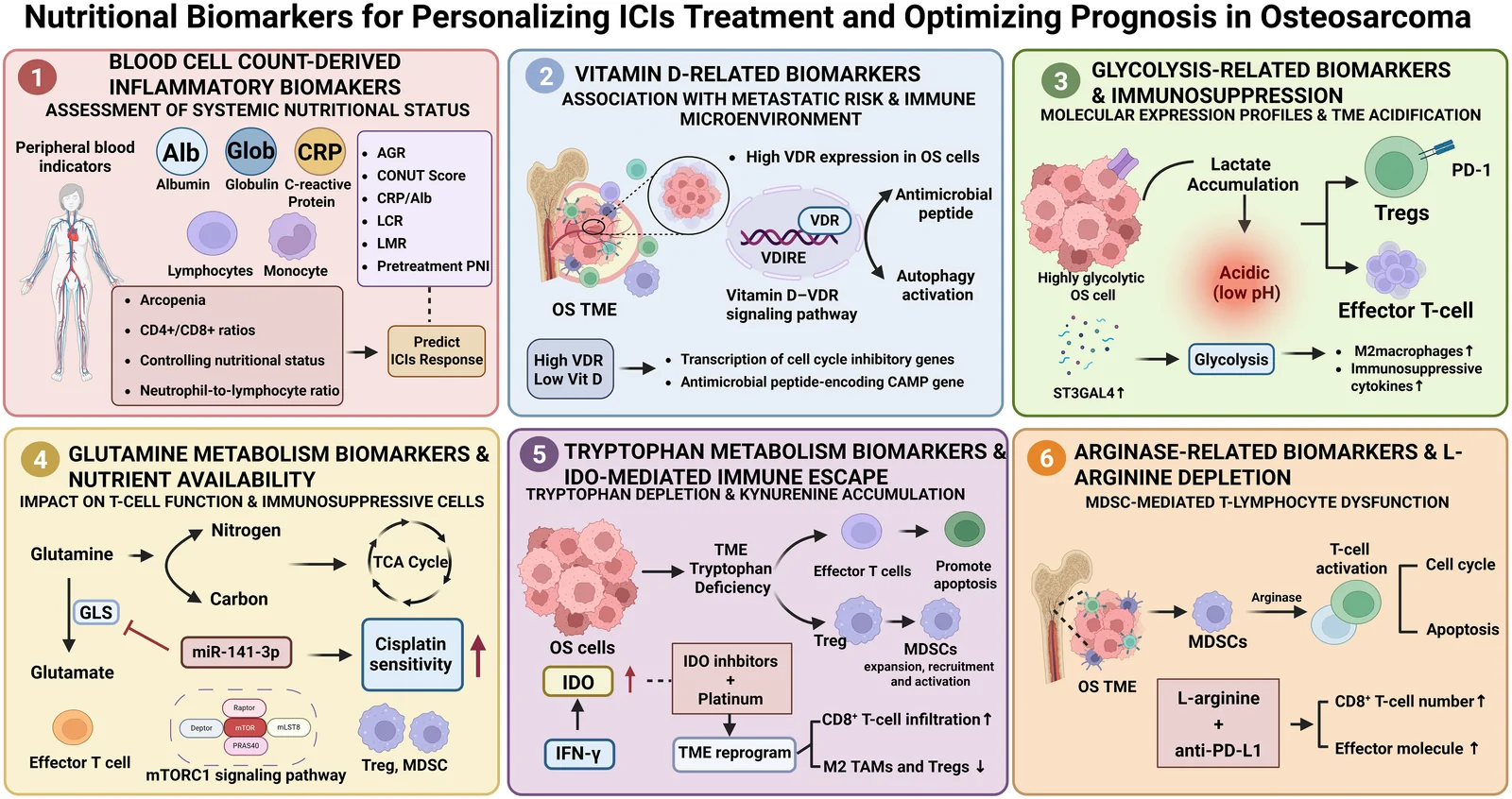
Nutritional and metabolic biomarkers for immunotherapy response in osteosarcoma. The diagram summarizes six biomarker categories—systemic nutritional and inflammatory indices, vitamin D, glycolysis, glutamine metabolism, tryptophan–IDO signaling, and arginase-mediated L-arginine depletion—and their effects on the osteosarcoma tumor immune microenvironment. These pathways regulate T-cell function, macrophage polarization, myeloid-derived suppressor cell activity, immune escape, and treatment resistance, thereby supporting patient stratification, prediction of immunotherapy response, and prognostic assessment. (Created in BioRender).

Nutritional biomarkers can be dynamically monitored through routine blood tests and may have important potential for predicting treatment responses and evaluating prognosis in patients with OS receiving ICIs. These biomarkers provide an integrated reflection of nutritional reserves, systemic inflammation, and immune status. Existing evidence primarily derives from prognostic analyses, immune microenvironment characterization studies, and molecular mechanism explorations, providing a basis for patient risk stratification and personalized treatment decisions. This section reviews the topic from three perspectives: the predictive value of serum nutritional biomarkers, the regulatory roles of molecular biomarkers, and clinical application strategies. Therefore, integrating nutritional biomarkers with tumor- and immune-related indicators may improve the accuracy of ICIs response prediction and prognostic stratification in OS (**Figure 2**).

### 3.1 Blood cell count-derived inflammatory biomarkers

Peripheral blood indicators serve as an important window reflecting the nutritional and metabolic status of OS patients and can provide reference for evaluating ICIs treatment response. One clinical study constructed a nutritional metabolism-related prognostic scoring system in a cohort of newly diagnosed OS patients, integrating nine indicators: albumin/globulin ratio, controlling nutritional status score, albumin, globulin, glucose, triglycerides, total cholesterol, high-density lipoprotein cholesterol, and low-density lipoprotein cholesterol (60). This score reflects patients’ nutritional reserves and metabolic reprogramming status. Although no single peripheral blood cell–related biomarker has yet been sufficiently validated as an independent predictor of overall survival in patients receiving immunotherapy, investigating peripheral blood–derived inflammatory and immune indices for predicting the efficacy of cancer immunotherapy remains an important area of clinical research. Peripheral blood lymphocytes are essential components of host cellular immunity and play a key role in mediating antitumor immune responses and reflecting systemic immune competence. The lymphocyte-to-C-reactive protein ratio (LCR) and lymphocyte-to-monocyte ratio (LMR) integrate information on lymphocyte-mediated antitumor immunity, systemic inflammation, and immunosuppressive status and are therefore considered promising clinical biomarkers. Previous studies have observed dynamic changes in LCR and LMR among patients treated with ICIs, and their baseline levels or treatment-related changes may be associated with therapeutic response and clinical outcomes (61). Accordingly, LCR and LMR may serve as simple, inexpensive, and readily available auxiliary indicators for assessing host immune status and predicting the efficacy and prognosis of immunotherapy, although their clinical utility requires further validation in large-scale prospective studies. Clinical studies have confirmed that indices such as preoperative LCR, albumin-to-globulin ratio(AGR), LMR, and C-reactive protein to albumin ratio (CAR) are significantly associated with survival outcomes in OS patients; some also demonstrate predictive value for response to neoadjuvant chemotherapy (62). These indices not only reflect patients’ systemic nutritional and inflammatory status but also indirectly correlate with ICIs treatment response by influencing host immune reserves and the degree of immunosuppressive microenvironment in the tumor. Studies in other solid tumors have demonstrated that PNI, arcopenia, CD4^+^/CD8^+^ ratios, controlling nutritional status (CONUT) and neutrophil-to-lymphocyte ratio(NLR), as key blood-derived biomarkers, are significantly associated with the efficacy of ICIs and patient survival; however, their predictive value in OS remains to be validated in future studies (63–67).

### 3.2 Vitamin D-related biomarkers

Vitamin D receptor(VDR) expression in osteosarcoma is associated with metastatic risk and immune microenvironment characteristics. Studies have found that high VDR expression in osteosarcoma patients with low serum vitamin D levels is significantly associated with metastasis (29). As a nuclear receptor transcription factor, VDR directly regulates the transcription of cell cycle inhibitory genes (such as p21 and p27) and various immune regulatory genes—including the antimicrobial peptide-encoding CAMP gene and genes involved in T-cell differentiation and cytokine regulation—by binding to vitamin D response elements (VDRE) (30, 68, 69). This influences osteosarcoma cell proliferative activity as well as T-cell infiltration and functional status in the tumor microenvironment. The vitamin D–VDR signaling pathway has been shown to regulate innate immune responses in monocytes and macrophages, including VDR expression, antimicrobial peptide production, and autophagy activation (70–73). On the basis of these immunomodulatory effects, vitamin D status may potentially influence the osteosarcoma immune microenvironment. Nevertheless, direct evidence regarding its effects on osteosarcoma cell proliferation, T-cell infiltration, macrophage polarization, and responses to ICIs remains lacking and requires validation in OS-specific experimental and clinical studies.

### 3.3 Glycolysis-related biomarkers

Glycolytic metabolic reprogramming is closely associated with the immune microenvironment of OS. Molecular classification based on glycolysis-related genes has revealed distinct immune infiltration patterns among osteosarcomas with different glycolytic states. Glycolysis may regulate immune-cell recruitment and function by altering nutrient competition, metabolite accumulation, and local microenvironmental conditions. Zhu et al. further developed a glycolysis-related prognostic model for patients with osteosarcoma comprising CHPF, RRAGD, TPR, and VCAN. This model not only stratified patients according to survival risk but was also associated with the immune microenvironment and drug sensitivity. Mechanistically, CHPF, RRAGD, and VCAN may indirectly influence glycolysis through AKT signaling, the nutrient-sensing-mTORC1 pathway, and extracellular matrix remodeling, respectively, whereas TPR may contribute to metabolic reprogramming through nucleocytoplasmic transport and the transcriptional regulation of metabolism-related genes (74). Lactate accumulation represents a potential mechanistic link between tumor glycolysis and immunosuppression. Cross-tumor studies have shown that lactate in highly glycolytic tumors can promote PD-1 expression in regulatory T cells and may impair effector T-cell function while favoring immunosuppressive myeloid-cell phenotypes. Meanwhile, meta-analyses in OS have demonstrated that elevated pretreatment serum LDH levels are associated with poorer event-free and overall survival (75, 76). Thus, LDH may be considered a metabolism-related prognostic marker worthy of further investigation, but its value as a predictive biomarker for immunotherapy in OS requires prospective validation. Wu et al. demonstrated that high ST3GAL4 expression promotes tumor cell glycolysis and drives polarization of tumor-associated macrophages toward the M2 phenotype, increases secretion of immunosuppressive cytokines, and shapes an immune escape state in the tumor microenvironment (16).

### 3.4 Glutamine metabolism related biomarkers

Glutamine metabolic reprogramming contributes to tumor metabolic adaptation by altering nutrient utilization and may affect immune-cell function within the tumor microenvironment (77). Glutamine supplies tumor cells with carbon and nitrogen for tricarboxylic acid cycle anaplerosis, biosynthesis, and redox regulation. Osteosarcoma studies have shown that highly metastatic osteosarcoma cells depend on glutamine to sustain proliferation and metabolic adaptation, and that GLS1 inhibition can restrict tumor growth and metastatic progression (78). YAP1-mediated glutamine metabolism compensates for the metabolic stress induced by ODC1 inhibition, whereas miR-141-3p mediated targeting of GLS suppresses glutamine metabolism and enhances cisplatin sensitivity (78–80).

Studies have shown that glutamine antagonism suppresses glycolytic and oxidative metabolism in tumor cells, alleviates hypoxia, acidosis, and nutrient depletion, and promotes compensatory metabolic adaptation in CD8^+^ T cells, thereby enhancing immune checkpoint blockade in selected tumor models (81). Glutamine metabolism contributes to remodeling of the tumor immune microenvironment by regulating mTORC1 signaling, autophagy (82). Glutamine deficiency significantly inhibits mTORC1 signaling pathway activation in effector T cells, reducing their glucose uptake, proliferative capacity, and IFN-γ secretion, while promoting recruitment and functional enhancement of MDSCs and regulatory T cells(Tregs), thereby exacerbating the immunosuppressive state of the tumor microenvironment (82–85). Targeting glutamine metabolism can restore glutamine availability in the tumor microenvironment, improve T-cell metabolic adaptability and cytotoxic function, and reduce infiltration of immunosuppressive cells, thereby indirectly enhancing the antitumor effect of immune checkpoint inhibitor therapy (81, 86).

### 3.5 Tryptophan metabolism related biomarkers

IDO protein, as a nutrition-related molecular biomarker, IDO primarily remodels the tumor immune microenvironment by regulating the tryptophan–kynurenine metabolic axis. On the one hand, IDO induces local tryptophan depletion within the tumor, leading to the accumulation of uncharged tRNAs in neighboring T cells and activation of the GCN2 pathway, which subsequently causes cell-cycle arrest and T-cell anergy. On the other hand, IDO promotes the accumulation of kynurenine and its downstream metabolites, thereby suppressing effector T-cell proliferation and inducing apoptosis. The combined effects of tryptophan depletion and kynurenine-derived metabolites can also promote the induction and activation of regulatory T cells. Therefore, elevated IDO expression in tumor cells can create an immune microenvironment conducive to tumor immune evasion by suppressing effector T cells and enhancing Treg-mediated immunosuppression (32). Tumor-derived IDO can promote the expansion, recruitment, and activation of MDSCs through a Treg-dependent mechanism, thereby inducing both local and systemic immunosuppression. Conversely, IDO inhibition can reduce the numbers of intratumoral MDSCs and Tregs and attenuate their immunosuppressive functions. Moreover, inflammatory cytokines within the tumor microenvironment, particularly IFN-γ, can induce IDO expression (87–89). IDO activity can serve as a molecular biomarker for stratifying OS patients for ICI therapy (89–93). Studies have shown that after PD-1 inhibitors relieve T-cell exhaustion, combination with IDO inhibitors can further restore tryptophan levels and reduce kynurenine-mediated immunosuppression, achieving dual reversal of immunosuppression and enhanced T-cell infiltration and killing function (94, 95). Farooq et al. emphasized IDO protein as a novel immunotherapeutic target in OS, whose inhibition can restore T-cell activity, suggesting that patients with high IDO protein expression are suitable for individualized regimens combining IDO inhibitors with ICIs (96).

Combination of IDO inhibitors with platinum (IV) prodrugs can reverse low immune responses in OS, increase CD8+ T-cell infiltration, and reduce M2-like tumor-associated macrophages and regulatory T cells in the TME to reprogram the microenvironment (97). For OS patients, detection of IDO biomarkers can serve as nutrition-related molecular markers to guide personalized treatment strategies and optimize ICI efficacy.

### 3.6 Arginase-related biomarkers

Arginase protein, as a nutrition-related molecular biomarker, regulates ICIs treatment prognosis in OS patients by consuming L-arginine. MDSCs-derived arginase protein modulates T-cell activation, thereby influencing cell cycle arrest and apoptosis (98, 99). OS mouse models have shown that L-arginine supplementation combined with anti-PD-L1 antibody can significantly increase CD8^+^ T-cell numbers and effector molecule protein expression, thereby prolonging survival (100).

Collectively, the available evidence suggests that nutritional biomarkers are not merely indicators of general nutritional status but may represent functional readouts of metabolic–immune interactions in OS. Peripheral inflammatory and nutritional indices reflect systemic immune reserves, whereas VDR expression and biomarkers related to glycolysis, glutamine, tryptophan, and arginine metabolism characterize distinct mechanisms of immune regulation. Mechanistically informed prospective studies and biomarker-guided clinical trials are therefore needed to determine whether targeting metabolic vulnerabilities can reverse immune suppression and enhance the efficacy of ICIs.

## 4 Conclusion

Nutritional biomarkers play a critical role in predicting ICIs efficacy and optimizing prognosis in osteosarcoma by regulating immune cell infiltration, metabolic reprogramming, and cytokine networks within the TME. Targeted nutritional interventions can remodel the TME and enhance antitumor immunity. Future prospective cohort studies are needed to validate the predictive performance of these biomarkers, integrate multi-omics data to construct individualized risk models, and explore nutrition–immunotherapy combination strategies to overcome treatment bottlenecks in OS and improve long-term patient benefit.
